# VHL Syndrome with Diabetes Mellitus, and Pulmonary and Thyroid Nodules: A Case Report

**DOI:** 10.15586/jkc.v12i3.412

**Published:** 2025-09-18

**Authors:** Zhiyuan Peng, Chuan Hua, Wenze Liu, Mingrui Zhou, Xiulan Yu, Yong Zhao, Xinhe Zuo

**Affiliations:** 1 Hubei University of Chinese Medicine, Wuhan, China;; 2 Hubei Provincial Hospital of Traditional Chinese Medicine, Wuhan, China

**Keywords:** diabetes mellitus, hereditary tumor syndrome, insulin therapy, Von Hippel–Lindau syndrome

## Abstract

Von Hippel–Lindau (VHL) syndrome is an autosomal dominant hereditary tumor syndrome caused by mutations in the *VHL* gene. It is characterized by the occurrence of tumors in multiple organs. Pancreatic involvement in VHL syndrome can present as pancreatic cysts or neuroendocrine tumors, which may interfere with both pancreatic exocrine and endocrine pancreatic functions. To our knowledge, no patients with VHL syndrome complicated by diabetes mellitus, pulmonary nodules, and thyroid nodules are reported in the literature. This study aims to explore the pathogenesis of diabetes, pulmonary nodules, and thyroid nodules in VHL syndrome through the analysis of a patient with VHL syndrome and to review relevant literature.

## Introduction

Von Hippel–Lindau (VHL) syndrome is an autosomal dominant hereditary tumor syndrome. It is caused by germline mutations in the *VHL* tumor suppressor gene located on the short arm of chromosome 3 (1, 2). The VHL protein is an E3 ubiquitin ligase that targets hypoxia-inducible factors (HIF), HIF-1a, HIF-2a, and HIF-3a, and is degraded by proteasome (3, 4). Under hypoxic conditions, the binding of VHL protein to HIF-1a and its degradation are inhibited, thereby allowing transcription of HIF-1 target genes to promote angiogenesis, proliferation, and metabolism (3). Loss of functional mutations in the *VHL* gene results in dysregulation of HIF pathway, with a significant increase in HIF-1 activity because of the impaired proteasomal degradation of HIF-1a and HIF-2a. Dysregulation of HIF promotes tissue-specific effects on cell growth and tumor susceptibility (5), leading to the development of tumors in multiple organs.

The reported prevalence of VHL is 1 in 36,000, with significant penetration in more than 90% of patients aged >65 years, and there are no clear reports that the prevalence and incidence of VHL are related to their ethnicity, race, culture, or gender. VHL syndrome is characterized by the presence of hypervascular tumors in multiple organs, including the central nervous system (CNS: cerebellum, brain stem, and spinal cord), retina, pancreas, adrenal gland, endolymphatic sac of the inner ear, epididymis (male), broad ligaments (female), and kidneys, among others (6, 7).

VHL syndrome has a characteristic genotype–phenotype correlation and is classified into three types. Type I VHL syndrome is defined by retinal and CNS hemangioblastomas, renal cysts/renal cell carcinomas, and pancreatic cysts, exhibiting a very low pheochromocytoma (PHEO) risk but high predisposition to hemangioblastomas and renal carcinomas. This subtype is predominantly caused by *VHL* gene mutations involving exon deletions, truncations, transitions, or nonsense variants that induce complete functional loss of VHL protein (pVHL). Patients are usually divided into 1A (with renal cell carcinoma) and 1B (without renal cell carcinoma) types according to the presence or absence of renal cell carcinoma. Type II, which includes pancreatic pheochromocytoma and islet cell tumors in addition to retinal and CNS angioblastomas, has a higher risk of PHEO and is characterized by VHL missense mutations (1, 2, 8), resulting in only single amino acid changes in VHL protein. Type II is subdivided into 2A (low-risk renal cell carcinoma [RCC]), 2B (high-risk RCC), and 2C (PHEO only) subtypes (8–10), according on the degree of risk of RCC. Type III is uncommon, and certain early-onset or highly aggressive forms of VHL may be included in this category, but there is no consensus about this. VHL-associated tumors frequently lose the functioning of the remaining wild-type VHL allele in a process known as loss of heterozygosity (LOH) (8, 9).

Pancreatic involvement in VHL syndrome can present as pancreatic cysts and neuroendocrine tumors, which may interfere with pancreatic exocrine and endocrine functions, leading to the development of diabetes mellitus (DM). However, clinical observations of VHL syndrome complicated by DM, pulmonary nodules, and thyroid nodules are extremely limited.

Through the analysis of the present patient, we explore the characteristics of VHL syndrome with diabetes, along with the formation of pulmonary and thyroid nodules. We collate the available data on the little-known association of VHL syndrome with significant dysglycemia with pulmonary nodules and thyroid nodules.

## Case Report

A 29-year-old male was admitted to the hospital in January 2021 because of unexplained weight loss over the past 6 months. He had lost approximately 7 kg of weight. In October 2020, during a routine physical examination, his fasting blood glucose level was found to be 9.2 mmol/L, and his urine glucose level was (+++). In December 2020, his fasting blood glucose level was rechecked at 10.1 mmol/L. Physical examination revealed a blood pressure of 132/80 mmHg, a pulse rate of 102 beats/min, height: 1.70 m, weight: 75 kg, and BMI: 25.95 kg/m^2^. No abnormalities were detected during cardiac or pulmonary examinations. The abdomen was soft, with no significant tenderness or rebound tenderness, and there was no edema in the lower limbs.

**Figure 1:**
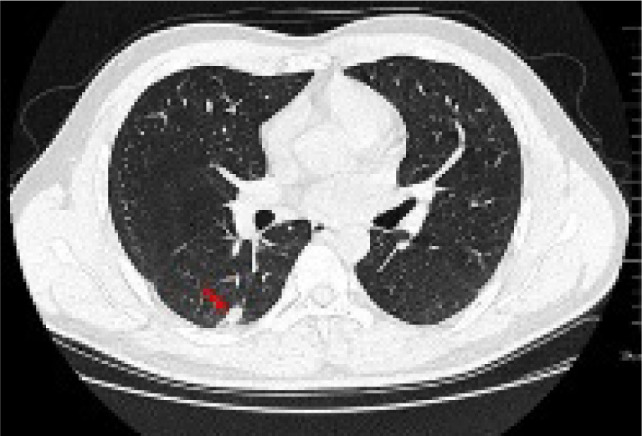
Lung CT shows that there is a calabash type density increase in the dorsal segment of the lower lobe of the right lung, the size of which was about 19×9 mm (red arrow).

Further examinations were conducted upon admission (Table 1). A chest CAT (CT) scan conducted on January 15, 2021 suggested a possible pulmonary infection with a shadow measuring approximately 19×9 mm in the right lower lobe (Figure 1).

**Table 1:** Laboratory measurements of the patient (2021, 2024).

	2021	2024	Normal range
ALT (U/L)	73	168	9–50
AST (U/L)	33	82	15–40
MAU (mg/L)	14.9	9.5	0–30
CREA (mmol/L)	53	43	57–97
INS (uIU/mL)	4.83	1.65	6–27
C-PE (ng/mL)	0.97	0.69	0.81–3.85
GLU (mmol/L)	8.6	20.6	3.9–6.1
KET (mmol/L)	0.1	0.4	0–0.3
HbA1C (%)	8.4	13.2	3.6–6.0
CEA (ng/mL)	3.48	7.07	0–5.0
AFP (ng/mL)	<1.3	<1.3	0–8.1
TP (g/L)	63.7	51.7	65–85
ALB (g/L)	38.6	35.2	40–55
Vit D (ng/mL)	6.83	6.07	<20 indicates deficiency
NSE (ng/mL)	27.89	9.98	<16.31
CYFRA21-1 (ng/mL)	No measure	3.14	<3.3
ACTH (pg/mL)	No measure	40.12	<46

ALT: alanine aminotransferase; AST: aspartate aminotransferase; MAU: microalbuminuria; CREA: serum creatinine; INS: insulin; C-PE: C-peptide; GLU: glucose; KET: ketone bodies; HbA1C: glycosylated hemoglobin, type A1C; CEA: carcinoembryonic antigen; AFP: alpha-feto protein; TP: total protein; ALB: albumin; Vit D: vitamin D; NSE: neuron-specific enolase; CYFRA21-1: cytokeratin 19 fragment; ACTH: adrenocorticotropic hormone.

**Figure 2:**
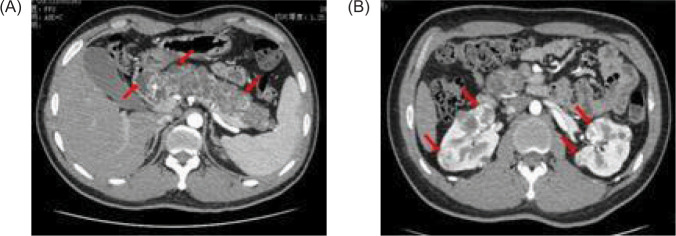
Contrast-enhanced CAT (CT) scan of the abdomen shows (A) multiple low-density nodules in the pancreas (red arrow); (B) low-density nodules in the left kidney, and multiple nodules of different sizes in both kidneys, which were heterogeneous enhancement (red arrow).

**Figure 3:**
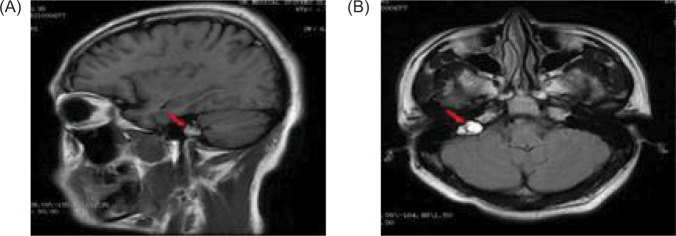
A magnetic resonance imaging (MRI) of the cranial reveals a possible mass in the right temporal bone jugular foramen, with a cross-sectional area of approximately 12×18 mm (red arrow); (A) sagittal position, and (B) flair view.

An abdominal CT on January 16, 2021 revealed renal and pancreatic masses, and an enhanced abdominal CT scan on January 18, 2021 showed multiple pancreatic cysts/cystadenomas and multiple kidney cancers in both kidneys and a small cyst in the left kidney (Figure 2). A cranial magnetic resonance imaging (MRI) done on January 29, 2021 revealed a possible mass in the right temporal bone jugular foramen, with a cross-sectional area of approximately 12×18 mm (Figure 3). The patient refused further examination of the brain and abdomen for personal reasons and denied any family history of VHL syndrome. He was diagnosed with DM and treated with tablet voglibose, 0.2 mg, three times daily (tds), and injection glargine insulin, 12 units once daily (OD). Despite the treatment, he continued to suffer from progressive weight loss over the next 6 months, losing an additional 7 kg.

In November 2024, the patient was readmitted to the hospital because of polydipsia, weakness in the lower limbs, and a weight loss of 38 pounds over the past 3 years. His current antidiabetic regimen included tablet acarbose, 50 mg, tds before meals. His fasting blood glucose levels ranged from 10 mmol/L to 14 mmol/L. Physical examination revealed a pulse rate of 95 beats/min, blood pressure of 124/87 mmHg, height: 1.71 m, weight: 56 kg, and BMI: 19.15 kg/m^2^. The patient appeared pale and fatigued, with no significant abnormalities detected during pulmonary or cardiac examinations, and no edema in the lower limbs. Laboratory examination was performed after admission (Table 1).

**Figure 4:**
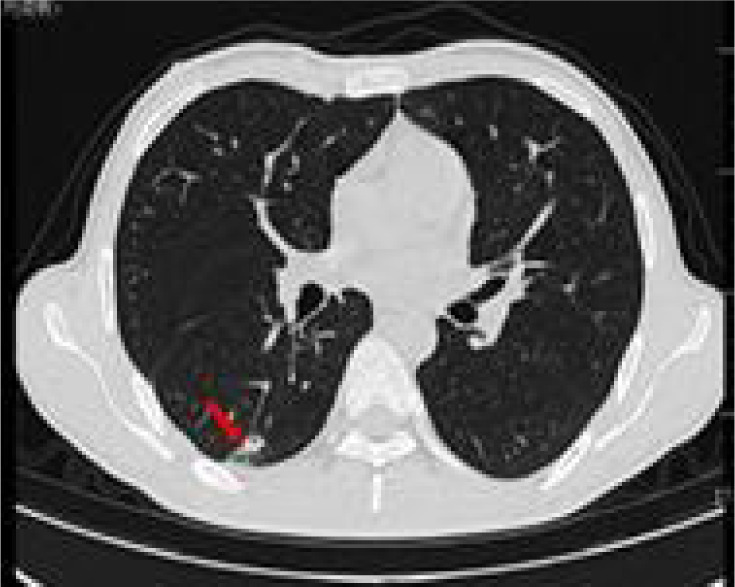
Lung CAT (CT) shows a ground glass nodule (12×9 mm) in the posterior basal segment of the right lower lobe (IM51), with signs of pleural depression (red arrow).

**Figure 5:**
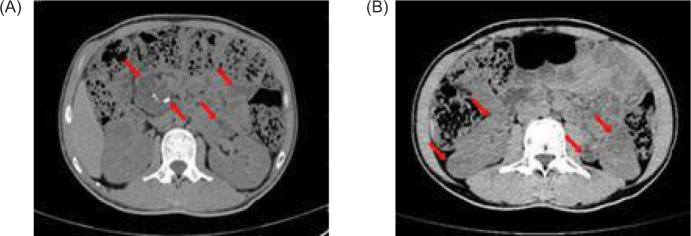
Full abdominal CAT (CT) scan shows pancreatic enlargement with (A) multiple cystic foci and scattered calcification in the pancreas (red arrow), and (B) multiple low-density nodules in the kidney (red arrow).

Full abdominal CT and lung CT revealed a high-risk pulmonary nodule in the right lower lobe (Figure 4), pancreatic enlargement with multiple cystic lesions, and multiple ill-defined low-density lesions in both kidneys (Figure 5).

**Figure 6:**
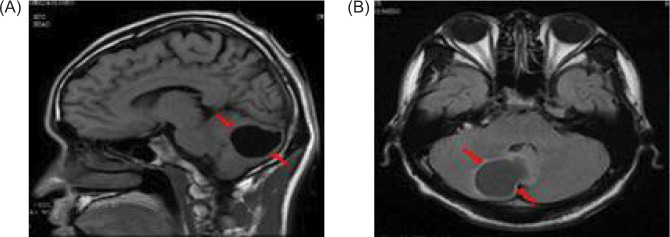
Magnetic resonance imaging (MRI) of the brain reveals a cystic mass in the right cerebellar hemisphere, approximately 40 mm in size (red arrow); (A) sagittal position, and (B) flair view.

**Figure 7:**
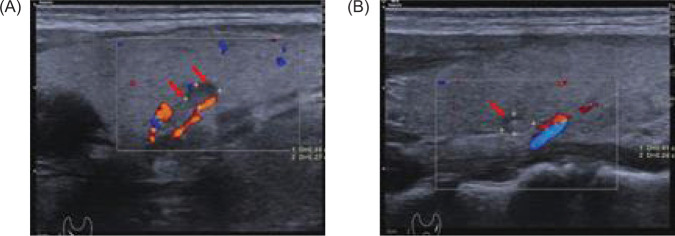
Thyroid color Doppler ultrasound shows bilateral cystic and solid thyroid nodules with clear boundaries, measuring (A) about 0.44×0.27 cm (right) (red arrow), and (B) 0.41×0.24 cm (left) (red arrow) (C-TIRADS: category 3).

Brain MRI revealed a cystic mass in the right cerebellar hemisphere measuring approximately 40 mm (Figure 6). Thyroid color Doppler ultrasound revealed solid and cystic nodules in both thyroid lobes (C-TIRADS: Category 3) (Figure 7). His fasting blood glucose level was 20.6 mmol/L. On November 29, 2024, he was treated with intensive insulin therapy, and his antidiabetic regimen was adjusted to injection aspart insulin, 4 units tds daily before meals and injection degludec insulin, 5 units once daily at night. The patient’s fasting blood glucose stabilized at 4.5–5.0 mmol/L, and postprandial blood glucose stabilized at 6.0–8.0 mmol/L in 3 days before discharge, and his clinical manifestations, such as polydipsia and weakness in the lower limbs, decreased significantly.

On December 18, 2024, genomic DNA was extracted from the patient’s blood sample to construct a genomic library. Target gene exons and adjacent splicing regions (approximately 20 bp), as well as the entire mitochondrial genome, were captured by probe hybridization and enriched. The enriched genes were subjected to quality control and sequenced using a next-generation sequencing instrument. A mutation in the *VHL* gene was detected. The raw sequencing data were first filtered to remove reads that did not meet quality control standards. The remaining reads were aligned with the human reference genome sequence (hg19) provided by the University of California Santa Cruz (UCSC) Genome Browser using genomic alignment software. Variants were identified using a mutation detection software module for single nucleotide variants (SNVs) and insertions/deletions (InDels). The variants were further annotated and filtered using professional databases, bioinformatics prediction software, and our own local databases and analysis software. Copy number variation (CNV) analysis was performed on the probe coverage regions using CNV detection algorithms.

A constitutional mutation in the *VHL* gene was detected (seq[GRCh37]del(3)(3p25.3)chr3:g.10183461_10183871del), indicating a possible copy number deletion variant of approximately 0.41 Kbp in the 3p25.3 region. This finding confirmed the diagnosis of VHL syndrome.

## Discussion

Von Hippel-Lindau syndrome comprises distinct subtypes associated with varied tumorigenic manifestations. In this case, the patient was diagnosed with VHL type 1A. Recent investigations (10), involving 58 VHL patients with hemangioblastomas, have revealed development of DM in six patients (10.3%). Notably, no documented patients with VHL syndrome concurrently presenting with DM, pulmonary nodules, and thyroid nodules exist. While pancreatic cysts in VHL may predispose to DM, this complication is not universally observed. Nonetheless, pancreatic neoplasms remain a plausible contributor to glucose dysregulation.

The pathogenesis of DM in VHL syndrome involves two primary mechanisms: (i) pheochromocytoma-induced secondary DM, and (ii) pancreatic endocrine dysfunction secondary to cystic replacement of parenchyma. In this case, the patient did not have pheochromocytoma but had pancreatic cysts, suggesting that the development of DM COULD be related to the extensive replacement of normal pancreatic tissue by cysts. VHL-related pancreatic pathology encompasses true cysts (serous cysts), serous cystadenomas, and neuroendocrine tumors (VHL-pNET) (11–14). Although pancreatic cysts typically remain clinically silent (11, 13, 15), emerging evidence associates extensive cystic involvement with DM pathogenesis (16–18). Few patients with DM associated with these pancreatic lesions are documented in the literature (Table 2). Current literature documents sparse patients with DM linked with such lesions (Table 2), which notably lack concomitant exocrine insufficiency—a distinguishing feature from classical type 3C DM. This suggests predominant endocrine impairment in VHL-related pancreatic disease. Supporting this hypothesis, a cohort study of 36 pancreatic tumor patients (19), identified DM in seven patients (19.4%), reinforcing the association between pancreatic neoplasia and metabolic dysregulation.

**Table 2:** Literature review of patients with VHL and diabetes and pulmonary nodule in the past 10 years.

Cases	Age/gender	Pancreas lesion	DM	IAA	Fasting C-peptide	Fasting insulin	Lung	Renal	CNS	PHEO	*VHL* gene mutation
Wang et al., 2024 (22)	28/female (F)	Neuroendocrine tumor	Y	NA	0.68	Not measured	N	N	N	Y	c.340+3_340+10delinsCG (in intron 1)
Wang et al., 2024 (22)	41/male (M)	Multiple cysts	Y	1.77	1.62	11.2	N	Y	Y	N	c.642+70C>A (in3’-untranslated region of exon 3)
Onishi et al., 2024 (19)	23/M	Neuroendocrine tumor	Y	NA	NA	NA	N	Y	Y	N	NA
Onishi et al., 2024 (19)	23/F	Neuroendocrine tumor	Y	NA	NA	NA	N	Y	Y	N	NA
Onishi et al., 2024 (19)	30/M	Pancreas cysts	Y	NA	NA	NA	N	N	Y	N	NA
Onishi et al., 2024 (19)	36/M	Pancreas cysts	Y	NA	NA	NA	N	N	Y	N	NA
Onishi et al., 2024 (19)	40/M	Neuroendocrine tumor	N	N	N	N	N	N	Y	N	NA
Onishi et al., 2024 (19)	41/M	Pancreas cysts	Y	NA	NA	NA	N	Y	Y	N	NA
Onishi et al., 2024 (19)	50/M	Neuroendocrine tumor	Y	NA	NA	NA	N	Y	Y	N	NA
Onishi et al., 2024 (19)	54/M	Pancreas cysts	N	N	N	N	N	Y	Y	Y	NA
Onishi et al., 2024 (19)	58/F	Neuroendocrine tumor	Y	NA	NA	NA	N	N	Y	N	NA
Huang et al., 2022 (17)	34/F	Multiple cysts	Y	NA	NA	NA	N	N	Y	N	c.233A>G(p.Asn78Ser)
Belaid et al., 2020 (23)	17/F	None	Y	NA	NA	NA	N	N	N	Y	c.191G>C(p.Arg64Pro)
Giri et al., 2020 (24)	34/F	Multiple cysts	Y	NA	NA	NA	N	N	Y	N	NA
Faiyaz-ul-Haque et al., 2020 (20)	44/M	Multiple cysts	N	N	N	N	Y	Y	Y	N	c.227_229delTCT(p.Phe76del)
Faiyaz-ul-Haque et al., 2020 (20)	45/M	Multiple cysts	N	N	N	N	N	Y	Y	N	c.227_229delTCT(p.Phe76del)
Saowapaet al., 2019 (25)	42/M	Multiple cysts	Y	NA	NA	NA	N	Y	N	N	c.263G>A(p.Trp88Stop)
Kaluarachchiet. al., 2018 (26)	34/F	None	Y	NA	NA	NA	N	N	Y	Y	No mutation detected
Ayloo et al., 2016 (27)	66/F	Multiple cysts	Y	NA	NA	NA	N	Y	Y	N	NA
Panayi et al., 2016 (28)	44/F	Neuroendocrine tumor, total pancreatectomy	Y	NA	NA	NA	N	Y	Y	N	NA
Heo et al., 2016 (29)	74/M	None	Y	NA	NA	NA	N	Y	Y	N	c.208G>A(p.Glu70Lys)
Medina et al., 2014 (30)	41/F	Serous cystadenoma	Y	NA	NA	NA	N	Y	N	N	NA
Present case	29/M	Multiple cysts	Y	NA	0.97	4.83	Y	Y	Y	N	seq [GRCh37] del(3) (3p25.3) chr3:g.10183461_ 10183871del

N: normal; Y: yes; NA: not acquired; DM: diabetes mellitus; IAA: insulin autoantibody; FCPE: fastig C-peptide; FINS: fatsing insulin; Renal: renal cysts or renal cancer; CNS: central nervous system hemangioblastomas; and PHEO: pheochromocytoma.

More rarely, this patient presented with pulmonary and thyroid nodules, which were not reported previously in the literature. A literature review revealed only two patients with VHL syndrome with multiple pulmonary nodules (20), but neither of these patients had DM. However, both patients (siblings) had the same germline mutation p.phe76del, which suggested that the pulmonary lesions in these patients were related to the specificity of the VHL protein mutation p.phe76del. Nevertheless, other diseases, such as pneumonia, pulmonary tuberculosis, and non-VHL-related tumors, can also cause pulmonary nodules. However, no significant respiratory manifestations, such as persistent dry cough, shortness of breath, chest pain, or pulmonary rales (crackles), were discovered in their clinical histories, indicating that VHL syndrome complicated by pulmonary nodules can be asymptomatic and easily overlooked in clinical practice, leading to potential underdiagnosis.

Regarding the pathogenesis of VHL syndrome complicated by thyroid nodules, studies have shown that *VHL* gene alterations, as a core molecular marker of metastatic clear cell renal cell carcinoma (ccRCC), have a key value in the diagnosis of thyroid nodules. Clear cell renal cell carcinoma (ccRCC), the most common malignancy with metastasis to the thyroid gland (>20%), is characterized by high frequency of *VHL* gene inactivation (49–89%), which is rare in primary thyroid tumors. Fine-needle aspiration (FNA) samples were examined by targeted sequencing of ThyroSeq v3. If VHL pathogenic mutations (e.g., missense/nonsense/frameshifting mutations) or copy number loss (CNA) were found together with loss of expression of thyroid epithelial markers (e.g., thyroglobulin), then it can specifically be diagnosed as metastatic ccRCC (all 18 cases were confirmed clinically). This strategy significantly addresses the cytologic dilemma—78% of metastatic ccRCC nodules are misclassified as Bethesda III (lesions of undetermined significance) because of morphologic overlap (e.g., microfollicular structures and intranuclear pseudoinclusions) or missing history (no history of renal cancer was reported in 28% of patients). Compared with immunohistochemistry (PAX8 was positive in both thyroid and renal cancers), VHL combined with thyroid markers could accurately identify metastases, especially in patients of advanced age (mean age: 68 years), male (male-to-female ratio: 2:1), multiple nodules (53%), or delayed metastasis after renal cancer surgery (maximum interval 18 years). This study provides a basis for early surgical intervention of solitary thyroid metastases (the median survival period is 21 months longer than untreated patients), but it should be noted that ThyroSeq does not cover VHL promoter methylation (~7% of ccRCC inactivation mechanisms) and some rare driver genes (such as *PBRM1*/*SETD2*) (21).

Although the pathogenesis of VHL syndrome complicated by DM, pulmonary nodules, and thyroid nodules has not been reported specifically, the present patient suggests that gene mutations in VHL syndrome not only lead to tumors in multiple organs but also affect various metabolic processes in the body, resulting in DM characterized by elevated blood glucose levels.

A comprehensive search was conducted on PubMed. A total of 133 studies were found by searching for “Von Hippel-Lindau syndrome” combined with “diabetes,” “glucose,” or “insulin” in the past 10 years. Additionally, one study was found for “Von Hippel-Lindau syndrome” combined with “pulmonary nodules,” one study for “Von Hippel-Lindau syndrome” combined with “thyroid nodules,” and no study for “Von Hippel-Lindau syndrome” combined with “diabetes,” “pulmonary nodules,” and “thyroid nodules.” A total of 22 eligible cases were reviewed (Table 2). Based on these reports, the relationship between VHL syndrome and DM, pulmonary nodules, and thyroid nodules is summarized as follows: (i) When VHL syndrome presents as pancreatic cysts, DM may occur; (ii) pancreatic DM may be associated with autoantibodies against insulin; (iii) in addition to pancreatic cysts, pheochromocytoma in VHL syndrome can also lead to DM; (iv) VHL patients with large pancreatic cysts are more likely to develop DM than those without pancreatic cysts; (v) pulmonary lesions in VHL syndrome may be related to the specificity of the VHL protein mutation p.phe76del; (vi) patients with VHL syndrome and pulmonary nodules usually do not have significant respiratory symptoms; and (vii) patients with VHL syndrome can simultaneously present with DM, pulmonary nodules, and thyroid nodules. Despite the presence of severe pancreatic lesions in the reported patient, his use of insulin degludec and the significant reduction in post-aspart blood glucose levels indicate a certain degree of compensatory potential within the remaining pancreatic tissue. Early clinical identification of pathogenic *VHL* gene variants in patients with VHL syndrome complicated by pulmonary and thyroid nodules helps in the detection and removal of tumors to prevent or reduce secondary complications.

## Conclusion

In summary, we present a case of VHL syndrome with DM associated with pulmonary and thyroid nodules. It was noted that the patient’s blood glucose levels were markedly elevated at the time of initial diagnosis of DM. However, following systematic treatment, patients with VHL syndrome were able to control rapidly and effectively their blood glucose using insulin therapy. This phenomenon may be attributed to the partial preservation of pancreatic function. This observation has significant clinical implications for the management of blood glucose concentrations in patients with VHL syndrome. Additionally, the presence of characteristic imaging features of pulmonary and thyroid nodules in this patient provides valuable insights into the diagnostic criteria for VHL syndrome.
